# Knowledge, Attitude, and Practice Towards Colorectal Cancer Prevention and Screening Among the Population in the Madina Region

**DOI:** 10.7759/cureus.99770

**Published:** 2025-12-21

**Authors:** Nada T Alharbi, Tariq A Alluqmani, Abdulelah N Alraddadi, Reyouf S Alsaedi, Atheer M Alahmadi, Abdulelah M Sinan, Mahmoud Abou-gamel

**Affiliations:** 1 Medicine, Taibah University, Medina, SAU; 2 Internal Medicine, Taibah University, Medina, SAU; 3 Family Medicine, Taibah University, Medina, SAU

**Keywords:** attitude, colorectal cancer, knowledge, madina, practice, saudi arabia, screening

## Abstract

Background

Colorectal cancer (CRC) is a major global health concern. Screening programs play a crucial role in its prevention. Following the COVID-19 pandemic, changes in health-seeking behavior and access to healthcare services may have influenced public awareness and adherence to CRC screening programs.

Objective

Our objective was to assess the level of knowledge and awareness regarding CRC prevention and screening among the population in Madinah, evaluate attitudes and practices related to CRC screening and identify barriers to adherence.

Methods

A cross-sectional study was conducted in the city of Madinah in Saudi Arabia and included 799 individuals aged 18 and above. The participants were randomly selected through a web-based questionnaire to obtain socio-demographic data, to assess the level of knowledge regarding risk factors and symptoms of CRC, and to evaluate the attitude and barriers to CRC screening among the population. Data were analyzed using descriptive statistics and inferential tests, including chi-square tests for associations between categorical variables. A p-value of <0.05 was considered statistically significant.

Results

The mean age of the participants was 28.5 ± 11.5 years, with the significant majority (86.1%) under the age of 45. Females constituted 77% while 67.3% of the study population achieved university-level education and only 3.9% reported a family history of CRC. Only 21% of the participants demonstrated good knowledge of CRC and its prevention methods, while the majority (57.8%) had poor knowledge. Variables such as being male, having university-level education, divorced, retired/unemployed or residents of outside Madinah were associated with poorer knowledge. Forty-two percent of the population got their knowledge about CRC from their friends, while only 10% received it through health care providers.

Conclusion

The study supports the need for further efforts to raise the awareness of society regarding CRC and its prevention and the necessity of applying clear health strategies and screening programs with collaboration of all health care facilities and providers within our health care system.

## Introduction

Colorectal cancer (CRC) refers to cancer originating in the colon or rectum, characterized by abnormal growth of cells in the lower digestive tract [1]. Globally, CRC is the third most common cancer after breast and lung cancers and the fourth leading cause of cancer-related mortality, with an estimated 0.69-0.88 million deaths annually [2-4]. In Saudi Arabia, CRC incidence gradually increased between 2000 and 2006, and it was more prevalent in men overall. CRC is the second most common cancer, ranking second in men and third in women [2,5]. Regional variations exist, with the Eastern Province reporting the highest incidence, followed by Riyadh, Makkah, Qassim, and Tabuk [2].

In Saudi Arabia, the five-year survival rate for colorectal cancer is 44.6%, lower than that of many countries, partly because patients often delay reporting symptoms [6]. Established risk factors include advanced age, male gender, inflammatory bowel disease, family history of CRC, presence of polyps, smoking, obesity, lack of exercise, low-fiber diet, and high red meat consumption [2,3,5]. Symptoms include altered bowel habits, abdominal pain, rectal bleeding, fatigue, and unexplained weight loss [3].

The United States Preventive Services Task Force (USPSTF) recommends CRC screening for adults aged 45-75 years [7]. Screening methods include colonoscopy every 10 years, flexible sigmoidoscopy every five years, or annual stool-based testing, with the fecal immunochemical test (FIT) now preferred over the traditional guaiac-based fecal occult blood test (gFOBT) in most current guidelines [8]. Despite these recommendations, uptake remains low worldwide. Previous studies in Saudi Arabia (Western region, Asir, Tabuk, Qassim, and Riyadh) have consistently shown poor public knowledge of CRC risk factors, symptoms, and screening [3,9-12]. In Madinah, a 2019 study similarly reported limited awareness of CRC [13].

This study aims to assess awareness and knowledge of factors related to CRC and its prevention among the population of Madinah City. This study will contribute updated data that can help identify current barriers and support health authorities in refining public health strategies. It also provides an opportunity to assess changes in awareness and behaviors following the COVID-19 pandemic and its impact on social and health practices. Additionally, this research will contribute to the development of focused initiatives to raise public awareness and understanding of colorectal cancer and screening, which will ultimately result in earlier diagnosis, better treatment results, and lower death rates.

The objectives of this study were to assess the population’s level of knowledge and awareness regarding CRC prevention and screening among the population of Madinah, to evaluate attitudes and practices related to CRC screening, and to identify the barriers to adherence with CRC prevention strategies and the factors associated with these barriers in current practice.

## Materials and methods

Study design

A descriptive cross-sectional study was conducted between 1 May and 31 July 2024 to assess the knowledge, attitude, and awareness regarding CRC screening among residents of Madinah, Saudi Arabia. Adults aged 18 years or older, regardless of gender or nationality, were eligible to participate if they resided in Madinah and provided consent. Individuals younger than 18 years, those who declined to participate, and those living outside Madinah were excluded from the study.

Sample size

The minimum sample size was calculated using the single proportion formula n = Z^2 p(1-p)/d^2, where Z = 1.96 for 95% confidence, p = 0.5 as a conservative estimate, and d = 0.05, yielding 385 participants. To increase precision and enable subgroup analyses, 799 participants were ultimately recruited, which reduced the maximum 95% margin of error to approximately 3.5% [14].

Data collection

A validated, self-administered questionnaire was adapted from two previous studies (Appendix) [1,6]. It was translated into Arabic and validated through a pretest involving 20 Arabic-speaking participants, including students and senior researchers. The pretest feedback from participants confirmed that the questionnaire was clear, comprehensible, and easy to complete. The electronic data collection links (Google Forms) were distributed to the target study population through WhatsApp, Telegram, and email. Consent was collected from the participants, which was the first question asked in the questionnaire, and the privacy of their information was ensured.

The questionnaire consisted of four sections. The first section obtained the participants’ sociodemographic data (age, sex, marital status, educational level, and occupation). The second section assessed their knowledge regarding risk factors, symptoms, and screening of CRC. The following sections evaluated the participants’ attitudes and practices toward CRC. The questionnaire was previously validated and pilot-tested on a small sample to ensure clarity and reliability before data collection.

Statistical analysis

Data analysis was conducted using SPSS Statistics version 22.0 (IBM Corp., Armonk, NY, USA). Descriptive statistics summarized the data, providing frequencies and percentages for categorical variables and mean and standard deviation for continuous variables. Knowledge about CRC and its screening was presented and tabulated for the study participants. This knowledge was evaluated through 13 questions, each with three possible responses: yes, no, and do not know. For simplicity, "no" and "do not know" responses were combined into a single "no" category. Knowledge levels were categorized as good, fair, and poor based on the respondents' scores: good knowledge was defined as over 75% correct answers, fair knowledge as 50-75%, and poor knowledge as less than 50% [15]. Chi-square tests were used to examine the association between knowledge levels and various participant characteristics, including age group (<45 vs. ≥45 years), sex (male vs. female), marital status (single, married, divorced/widowed), educational level (secondary vs. university), occupation (student, employed, retired), place of residence (Madinah city vs. other cities), and family history of CRC among first-degree relatives (yes vs. no). Attitudes toward CRC and its screening methods were assessed using four Likert-scale items (agree, neutral, disagree). A p-value ≤ 0.05 was considered statistically significant. Additionally, awareness and recognition of symptoms associated with CRC and causes of not following CRC screening program were assessed and tabulated.

Ethical approval was obtained from the Research Ethics Committee of Taibah University (approval TU-IRB-2024-CR-015). Detailed informed consent was provided to all participants, outlining the study’s purpose and confirming that participation was entirely voluntary. All collected data were securely stored and used solely for research purposes.

## Results

A total of 799 participants from the Madinah region were included in the analysis. The mean age was 28.5 ± 11.5 years (range: 18-67 years). The majority of participants were younger than 45 years (688 [86.1%]), while 111 (13.9%) were 45 years or older. Most participants were female (615 [77.0%]) and single (518 [64.8%]). Married individuals accounted for 257 (32.2%), while 24 (3.0%) were divorced or widowed. Regarding educational attainment, 538 (67.3%) had university-level education and 261 (32.7%) had secondary education. Students represented the largest occupational group (426 [53.3%]), followed by employed participants (331 [41.4%]) and unemployed/retired individuals (42 [5.3%]). Only 31 (3.9%) reported a first-degree family history of CRC. The majority of respondents resided in Madinah city (731 [91.5%]), while 68 (8.5%) lived outside the city (Table 1).

**Table 1:** Demographic characteristics of the subjects Data are presented as mean ± SD and n (%) **Including Yanbu, Al-Ala, El-Hankia CRC: colorectal cancer

Characteristic	Category	n (%)
Age (years, mean ± SD, range)	–	28.5 ± 11.5 (18–67)
Age group	< 45 years	688 (86.1)
	≥ 45 years	111 (13.9)
Sex	Male	184 (23.0)
	Female	615 (77.0)
Marital status	Single	518 (64.8)
	Married	257 (32.2)
	Divorced/Widowed	24 (3.0)
Education level	Secondary	261 (32.7)
	University	538 (67.3)
Occupation	Student	426 (53.3)
	Employed	331 (41.4)
	Unemployed/Retired	42 (5.3)
Family history of CRC	Yes	31 (3.9)
	No	768 (96.1)
Place of residence	Madinah City	731 (91.5)
	Outside Madinah	68 (8.5)

**Figure 1:**
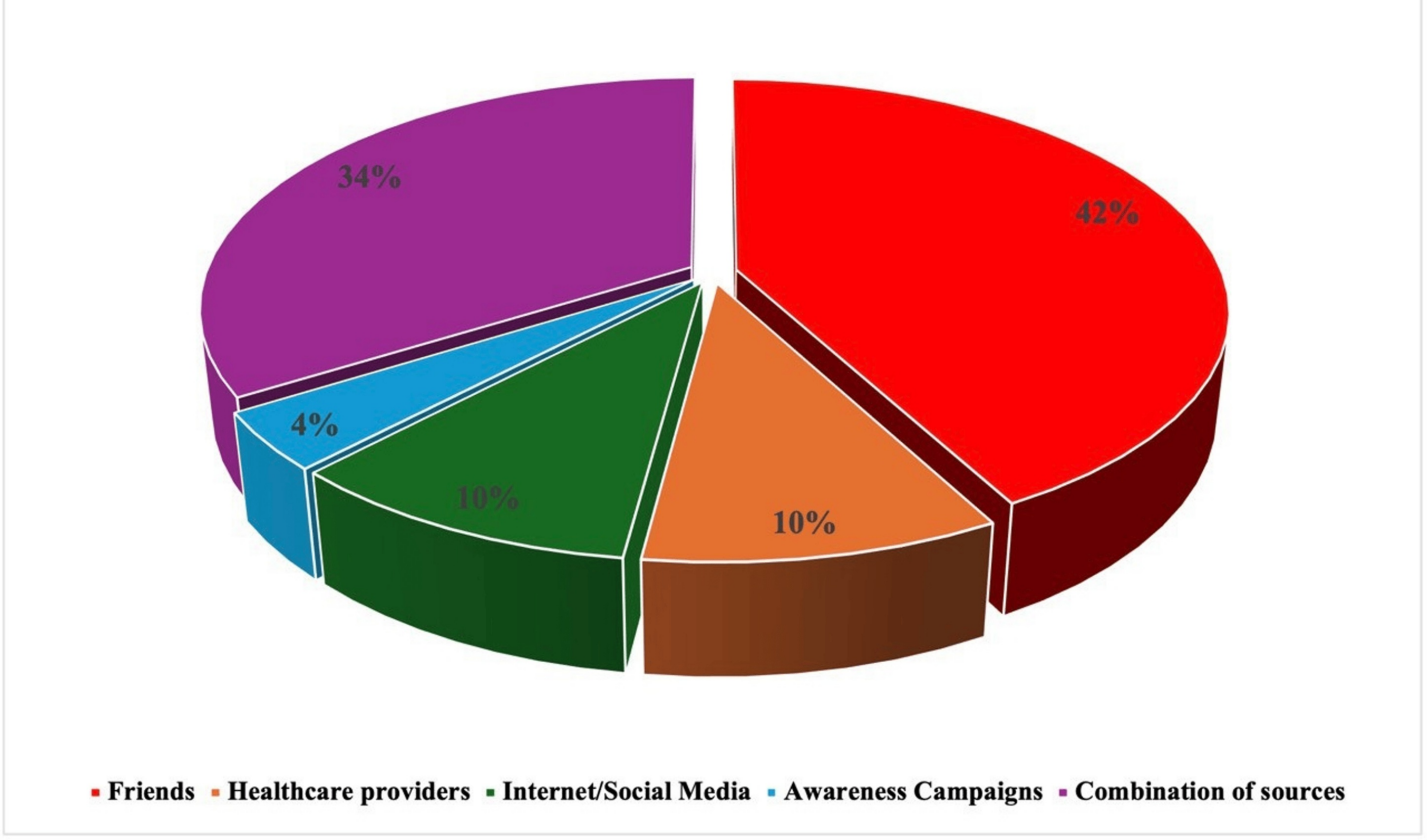
Distribution of the studied 799 subjects by their source of information about colorectal cancer (CRC)

**Figure 2:**
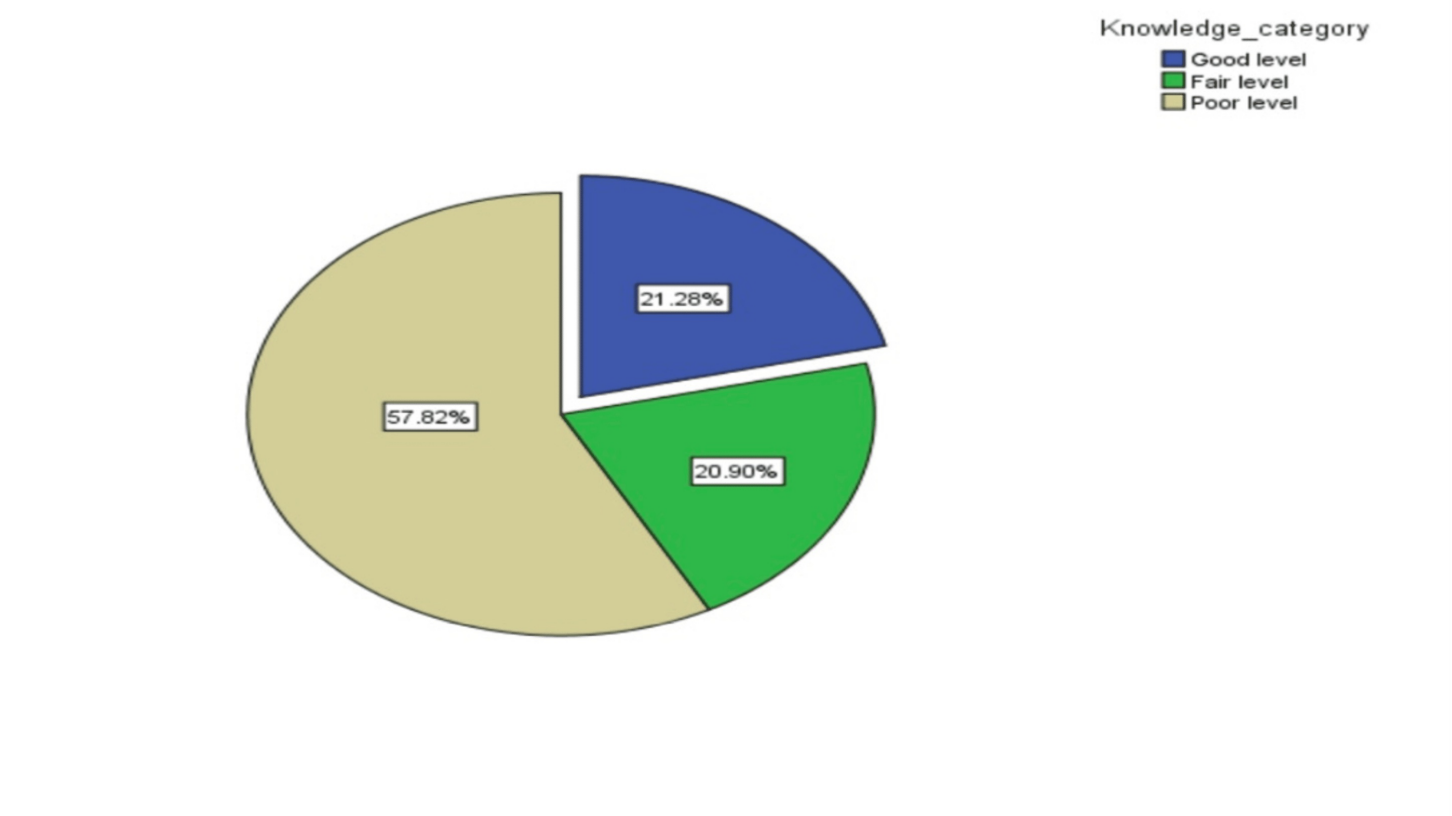
Percent distribution of the studied 799 subjects by level of their knowledge about colorectal cancer (CRC)

Table 2 presents a comprehensive overview of the knowledge among the studied subjects regarding CRC. A notable finding is that the majority of participants believe that factors such as family history (578 [72.3%]), alcohol consumption (581 [72.7%]), lack of exercise (448 [56.1%]), and smoking (444 [55.6%]) increase the risk of developing CRC. In contrast, fewer participants were aware of the risks associated with red meat consumption (313 [39.2%]) or older age (394 [49.4%]). Notably, only 72 (9.0%) correctly identified the recommended age (45 years) for initiating CRC screening. The sources of CRC information reported by participants are shown in Figure 1. Friends were the most common source (42%), followed by healthcare providers (10%), social media and the internet (10%), and awareness campaigns (4%). About one-third (34%) used multiple sources of information. The distribution of knowledge levels is shown in Figure 2. Only 170 (21.3%) demonstrated good knowledge, while 167 (20.9%) had fair knowledge and 462 (57.8%) had poor knowledge.

**Table 2:** Distribution of the studied subjects by their knowledge about colorectal cancer (CRC)

Knowledge items	Answer n (%)		
	Yes	No	Do not know
Does colon cancer most often occur due to a person's behavior or lifestyle?	441 (55.2%))	61 (7.6%))	297 (37.2%))
Consuming a low amount of fruits and vegetables increases the risk of developing colon cancer.	348 (43.6%))	232 (29.0%))	219 (27.4%))
Lack of exercise increases the risk of developing colon and rectal cancer?	448 (56.1%))	106 (13.2%))	245 (30.7%))
Weight gain increases the risk of developing colon and rectal cancer?	481 (60.2%))	63 (7.9%))	255 (31.9%))
A family history of colon and rectal cancer increases the risk of developing colon and rectal cancer?	578 (72.3%))	49 (6.1%))	172 (21.5%))
Are smokers at a higher risk of developing colon and rectal cancer?	444 (55.6%))	78 (9.7%))	277 (34.7%))
Does alcohol consumption increase the risk of developing colon and rectal cancer?	581 (72.7%))	34 (4.3%))	184 (23.0%))
1. Does consuming a lot of red meat increase the risk of developing colon and rectal cancer?	313 (39.2%))	99 (12.4%))	387 (48.4%))
2. Are older adults at a higher risk of developing colon and rectal cancer?	394 (49.4%))	93 (11.6%))	312 (39.0%))
3. Are individuals with irritable bowel syndrome at a higher risk of developing colon and rectal cancer?	355 (41.9%))	78 (9.8%))	386 (48.3%))
4. Are men at a higher risk of developing colon and rectal cancer?	309 (38.7%))	87 (10.9%))	403 (50.4%))
5. Does colon cancer worsen over several years? 6.	560 (70.1%))	16 (2.0%))	223 (27.9%))
7. Based on health recommendations, when does a person typically start screening for colon and rectal cancer for prevention? Soon after turning 45 years.	72 (9.0%))	350 (45.1%))	377 (45.9%))

Associations between knowledge levels and sociodemographic characteristics are presented in Table 3. Knowledge level was significantly associated with gender (p = 0.02), with a higher proportion of females falling into the good-knowledge category compared with males (139 [22.6%] vs. 31 [16.8%]). A significant association was also observed between knowledge level and marital status (p < 0.001), with single participants showing a higher proportion of good knowledge (139 [26.9%]) compared with married (29 [11.3%]) and divorced/widowed individuals (two [8.3%]). Occupation was similarly associated with knowledge level (p < 0.001), as students represented the largest proportion of participants with good knowledge (122 [28.8%]), relative to employed (43 [13.0%]) and unemployed/retired respondents (five [11.9%]). Place of residence also showed a significant association (p = 0.02), with a higher proportion of Madinah residents classified as having good knowledge (163 [22.3%]) compared with those living outside the city (seven [10.3%]). In contrast, age group (p = 0.35) and family history of CRC (p = 0.50) were not significantly associated with knowledge levels.

**Table 3:** Distribution of the studied subjects according to their level of knowledge about colorectal cancer (CRC) and their characteristics Data are presented as n (%). The Chi-square test was used. A p-value < 0.05 was considered statistically significant. Age is measured in years. “-” indicates not applicable.

Characteristic	Good n (%)	Fair n (%)	Poor n (%)	p-value
Age				0.35
<45 years	152 (22.1)	141 (20.5)	395 (57.4)	—
≥45 years	18 (16.2)	26 (23.4)	67 (60.4)	—
Gender				0.02*
Male	31 (16.8)	31 (16.8)	122 (66.3)	—
Female	139 (22.6)	136 (22.1)	240 (55.3)	—
Marital status				<0.001
Single	139 (26.9)	109 (21.0)	270 (52.1)	—
Married	29 (11.3)	51 (19.8)	177 (68.9)	—
Divorced/Widowed	2 (8.3)	7 (29.2)	15 (62.5)	—
Education level				<0.001
Secondary education	75 (28.5)	58 (22.5)	128 (49.0)	—
University education	95 (17.7)	109 (20.3)	334 (62.1)	—
Occupation				<0.001
Student	122 (28.8)	93 (21.8)	211 (49.4)	—
Employed	43 (13.0)	69 (20.8)	219 (66.2)	—
Unemployed/Retired	5 (11.9)	5 (11.9)	32 (76.2)	—
Family history of CRC				0.5
Yes	4 (12.9)	7 (22.6)	20 (64.5)	—
No	166 (21.6)	160 (20.8)	442 (57.6)	—
Place of residence				0.02*
Madinah City	163 (22.3)	156 (21.3)	412 (56.4)	—
Outside Madinah	7 (10.3)	11 (16.2)	50 (73.5)	—

**Figure 3:**
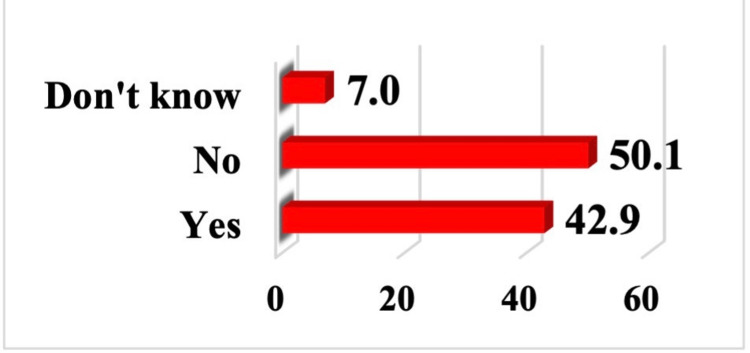
Percent distribution of the study subjects by their hearing about colorectal cancer (CRC) screening program

**Figure 4:**
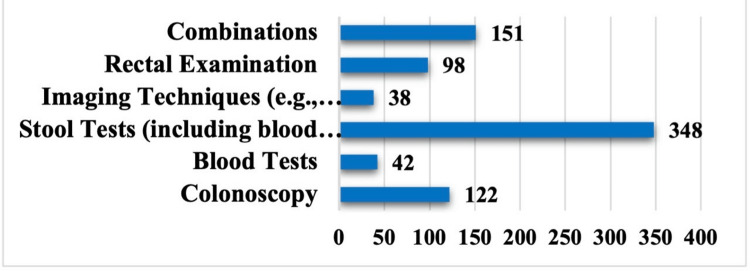
Distribution of the study subjects by their answers regarding the appropriate method for early detection of colon cancer

Table 4 presents the frequency distribution of attitudes among the studied subjects toward CRC and its screening. A significant majority of participants (536 [67.1%]) agree with the statement that it is important for them to know about colon and rectal cancer. (585 [73.2%]) of the study population agree with the notion that early-stage CRC can be treated more effectively, while 285 (35.7%) of the studied subjects disagree regarding the efficacy of CRC screening tests. Additionally, although 219 (27.4%) disagree that cancer screening should be implemented on a large scale, a substantial majority, 501 (62.8%), agrees. Awareness of the studied subjects about the CRC program and the best CRC screening test modality are depicted in Figures 3, 4.

**Table 4:** Distribution of the studied subjects regarding their attitude toward colorectal cancer (CRC) and its screening

Attitude items	Agree N (%)	Neutral N (%)	Disagree N (%)
It is important for me to know about colon and rectal cancer.	536 (67.1%))	45 (5.6%))	218 (27.3%))
Colon and rectal cancer diagnosed at an early stage can be treated more effectively.	585 (73.2%))	42 (5.3%))	172 (21.5%))
Colon and rectal cancer screening tests are effective.	394 (49.3%))	120 (15.0%))	285 (35.7%))
Cancer screening should be implemented on a large scale	501 (62.8%))	79 (9.9%))	219 (27.4%))

Awareness of CRC symptoms is summarized in Table 5. Blood in stool was identified by 120 (15.0%) participants, sudden weight loss by 80 (9.9%), abdominal pain and cramps by 63 (7.9%), and incomplete rectal emptying by 78 (9.8%). Changes in bowel movements were recognized by 49 (6.1%). The most frequently reported response was a combination of symptoms, including blood in stool, sudden weight loss, and mucus, cited by 409 (51.1%).

**Table 5:** Awareness and recognition of symptoms associated with colorectal cancer (CRC) among the study subjects

Symptoms	n (%)
Blood in Stool	120 (15.0%))
Sudden Weight Loss	80 (9.9%))
Abdominal Pain and Cramps	63 (7.9%))
Changes in Bowel Movements	49 (6.1%))
Incomplete Rectal Emptying	78 (9.8%))
Combination of symptoms: including blood in stool, sudden weight loss, and mucus.	409 (51.1%))

The barriers to CRC screening are summarized in Table 6. The most frequently reported reason for avoiding screening was a combination of factors such as fear, embarrassment, lack of information, or time constraints (458 [57.3%]). Other barriers included lack of symptoms (139 [17.4%]), fear or embarrassment alone (97 [12.1%]), lack of information (67 [8.4%]), and time constraints (23 [2.9%]). A small number of participants (15 [1.9%]) reported other unspecified reasons.

**Table 6:** Causes of avoiding colorectal cancer (CRC) screening among the studied subjects

Causes	n (%)
Fear or Embarrassment	97 (12.1%))
Lack of Symptoms	139 (17.4%))
Lack of Information	67 (8.4%))
Time Constraints	23 (2.9%))
Combination of Factors	458 (57.3%))
Other unspecified causes	15 (1.9%))

## Discussion

CRC is one of the most common malignancies, particularly in Saudi Arabia, and places a substantial burden on the healthcare system. Efforts continue at both international and national levels to educate the population and provide reliable screening programs to aid in prevention and early detection of this disease.

In our study, when assessing the overall knowledge level regarding CRC, 170 participants (21%) demonstrated good knowledge, while the majority, 462 participants (57.8%), had poor knowledge. Participants were relatively knowledgeable about CRC risk factors, including family history (578 participants, 72.3%), alcohol consumption (581 participants, 72.7%), and lack of exercise (448 participants, 56.1%). However, they had less awareness about the effect of diet on CRC risk, as well as a poorer understanding of demographic risk factors. Regarding CRC screening, only 72 participants (9%) were able to identify the correct age to start screening, while 342 participants (42.9%) reported that they had heard about CRC screening programs.

Upon questioning about reasons for avoiding screening, a combination of factors was the most evident; these included fear or embarrassment (97 participants, 12.1%), lack of symptoms (139 participants, 17.4%), and time constraints (23 participants, 2.9%).

Three hundred four participants (38%) had heard about CRC screening, but only 105 participants (13.1%) correctly identified the appropriate age to begin, with many mistakenly believing it should start before the age of 50. Participants in that study demonstrated slightly better awareness of dietary risk factors - such as the impact of consuming red meat and having a low intake of fruits and vegetables - compared to our participants. Nevertheless, both studies reported similar barriers to undergoing screening, primarily including fear, embarrassment, lack of symptoms, and limited time [13]. The difference in awareness, particularly regarding dietary risk factors, may be due to variations in participant demographics, survey design, or timing.

In Al-Ahsa, Saudi Arabia, awareness about CRC was assessed among teachers through a self-administered questionnaire. This study revealed almost similar results, where the majority had inadequate knowledge about CRC risk factors. The majority were also unaware of CRC screening tests, despite a positive family history in about 12% of participants, yet higher educational levels were associated with better awareness [16]. In Riyadh, Saudi Arabia, a study conducted by Alshammari et al. among participants aged above 40 years demonstrated that less than half had heard about CRC screening, and only 6% had actually undergone screening [6,17].

Relatively different results were found among students’ family members in China, who demonstrated good knowledge scores regarding CRC risk factors. More than half of them demonstrated positive attitudes toward screening programs. Higher knowledge, positive attitudes, and better practices were evident among females, as well as among people with positive family history and higher education. Another Chinese study assessed CRC knowledge among the elderly population (50-75 years) and demonstrated good knowledge, positive attitude, and better practices regarding CRC and its screening [18-20].

In the healthcare setting, CRC knowledge was assessed among medical students, community pharmacists, and PHC physicians in Saudi Arabia. Students showed low knowledge and poor attitudes toward CRC screening; literature recommends starting education earlier in medical school to enhance the knowledge of future physicians [5,21].

In our study, several sociodemographic factors were significantly associated with participants’ level of knowledge about CRC. Female participants had significantly higher knowledge levels than males (139 (22.6%) vs. 31 (16.8%), P = 0.02), which aligns with the findings of Alsayed et al. (2019) [13]. Single individuals showed significantly higher knowledge levels than those who were married or previously married (139 (26.9%) vs. 29 (11.3%) vs. two (8.3%), P < 0.0001). Participants with secondary education demonstrated better awareness than university-educated individuals (75 (28.5%) vs. 95 (17.7%), P < 0.0001), and students were the most knowledgeable among all occupational groups (122 (28.8%), P < 0.0001). Several factors may explain this pattern. University-educated individuals may rely more heavily on online and informal digital sources rather than structured health education, which may expose them to inconsistent or incomplete information. Another possible explanation relates to sampling imbalance, as the study recruited disproportionately more university-educated participants, which may have influenced the distribution of knowledge scores. Awareness was also higher among residents of Madinah city compared to those living outside it (163 (22.3%) vs. seven (10.3%), P = 0.02).

Sources of information used by our participants included friends, healthcare providers, and social media. Participants’ characteristics affecting knowledge levels included gender, residence, and occupation, while family history of CRC had no significant effect.

Regarding participants’ attitudes toward CRC and its screening, 536 participants (67%) agreed that it is important to know about CRC, and the majority indicated that early detection of CRC would improve treatment outcomes. In comparison, cancer survivors surveyed in other studies demonstrated higher knowledge of CRC symptoms.

Regarding CRC screening, about half of patients underwent screening based on physician recommendation, whereas those who did not cited lack of symptoms or no physician recommendation as primary reasons [22,23]. In our study, only 343 participants (42.9%) reported having heard of CRC screening programs, while the majority either responded negatively (401 participants, 50.1%) or were unsure (56 participants, 7%) (Figure 3). Stool-based tests were the most recognized screening method, followed by colonoscopy and rectal exams, with few participants identifying multiple methods. Similarly, in the Surrati study, most participants lacked awareness of standard screening tools, reflecting a shared gap in understanding effective CRC screening approaches [13].

The most common barrier to CRC screening in our study was a combination of factors, mainly lack of symptoms, fear or embarrassment, and limited information. This aligns with Surrati et al., who also reported emotional and practical barriers as major obstacles, highlighting the persistent role of perceived risk and discomfort in avoiding screening [13].

Limitations of our study include the use of a self-administered, web-based questionnaire, which may introduce both reporting and selection bias. Although the questionnaire was adapted from validated sources and supported by clear instructions to minimize response errors, the convenience sampling strategy-primarily distributed through social media-likely resulted in a demographically skewed sample. The study population consisted predominantly of young, female, and university-educated participants, which may limit the generalizability of the findings to the broader community, particularly older adults who are the primary target group for colorectal cancer screening. Additionally, while the questionnaire was adapted from previously validated studies, the internal consistency (Cronbach’s alpha) and content validity (CVI/CVR) of the Arabic version were not formally assessed. Consequently, the results should be interpreted within the context of the sampled population, and these limitations should be considered when applying the findings to public health planning or future research.

## Conclusions

In conclusion, the study indicates that knowledge, attitudes, and practices related to CRC and its screening were suboptimal among the surveyed participants, including younger and highly educated groups where higher awareness might typically be expected. Although awareness appears slightly improved compared with earlier reports, notable gaps remain.

These findings should be interpreted with caution given the study’s limitations, particularly the convenience sampling and demographic skew toward younger, female, and highly educated individuals, which restrict generalizability to the wider Madinah population - especially the 45-75 age group targeted for CRC screening. 

Despite these limitations, the results highlight the need for improved educational initiatives, culturally tailored awareness efforts, and enhanced communication of screening guidelines. Addressing common barriers such as fear, embarrassment, and lack of perceived symptoms remains essential. Healthcare providers can further support awareness by integrating CRC education into routine clinical encounters.

## Appendices

Questionnaire

**Table 7:** Demographic characteristics of the participants English version modified from [1,6] and translated into Arabic by the authors.

1-Sex: Male Female 2-Age: 3-Marital status: single Married Divorced widowed 4-Education level: general education University education high education 5-From any of the city’s governorates AL Madinah AL Munawwarah Al-Ula Al-Ais It stems Cradle Alhanakiya 6- occupation: students unemployed retired government employee private sector employee 7--work nature: 1-Field 2-Practical 8- family members (son,daughter,brother,sister) or close friends had a Colorectal cancer history: yes, no If yes who: -son -daughter -brother -sister	1-الجنس: ذكر أنثى 2-العمر: 3-الحالة الاجتماعية: أعزب متزوج مطلق ارمل 4-المستوى التعليمي: تعليم عام تعليم جامعي تعليم متقدم (ما بعد البكالوريوس) من أي محافظات المدينة5- المدينة المنورة العلا العيص ينبع المهد الحناكية 6- المهنة : طالب غير موظف متقاعد موظف حكومي موظف في القطاع الخاص 7-طبيعة العمل: ١-ميداني ٢- مكتبي 8- أصدقاء او احد الاقارب المقربين من الدرجة الاولي(اب .ام ، اشقاء ، آبناء)لديهم تاريخ سرطان القولون والمستقيم: نعم / لا اذا نعم من: ابن ابنة اخ اخت

**Table 8:** Knowledge items about colorectal carcinoma English version modified from [1,6] and translated into Arabic by the authors.

1-Is colon cancer most often caused by a person’s behavior or lifestyle? Yes No I don’t know 2-Do people eating a low-fat and high-fibre diet seem to have a lower risk of colon cancer? Yes No I don’t know 3- Do people who do not exercise seem to have a high risk of colorectal cancer? Yes No I don’t know 4-Do people who are overweight tend to have a high risk of colorectal cancer? Yes No I don’t know 5- Do people who have a family history of colorectal cancer tend to have a high risk of colon cancer? Yes No I don’t know 6- Do smokers have a high risk of colorectal cancer? Yes No I don’t know 7-Do people who drink alcohol tend to have a high risk of colorectal cancer? Yes No I don’t know 8- Do people who eat a lot of red meat tend to have a high risk of colorectal cancer? Yes No I don’t know 9- Do people of old age tend to have a high risk of colorectal cancer? Yes No I don’t know 10- -Are men more susceptible to colorectal cancer? Yes No I don’t know 11- Do people who have irritable bowel syndrome tend to have a high risk of colorectal cancer? Yes No I don’t know 12- what are Symptoms of colon cancer? The presence of blood in the stool Sudden weight loss Increase mucus secretions in the stool Feeling that the rectum was not fully emptied with defecation Pain and cramps in the stomach Sudden change in number of bowel motions and Diarrhea 13-What are methods of early detection of colon cancer? Colonoscopy PR speculum Blood detection in the stool sample Barium dye for large intestine Blood tests Abdominal CT scan Clinical examination of the rectum 14--When does a person begin the examination? When symptoms appear From the age of 35-40 40-50 50-60	1-هل يحدث سرطان القولون في أغلب الأحيان بسبب سلوك الشخص أو أسلوب حياته؟ نعم لا لا أعرف 2- هل يبدو أن الأشخاص الذين يتناولون نظامًا غذائيًا قليل الدهون وعالي بالالياف لديهم خطر أقل للإصابة بسرطان القولون؟ نعم لا لا أعرف 3- هل يبدو أن الأشخاص الذين لا يمارسون الرياضة لديهم خطر اعلى للإصابة بسرطان القولون والمستقيم؟ نعم لا لا أعرف 4- هل الأشخاص الذين يعانون من زيادة الوزن يميلون إلى زيادة خطر الإصابة بسرطان القولون والمستقيم؟ نعم لا لا أعرف 5- هل الأشخاص الذين لديهم تاريخ عائلي للإصابة بسرطان القولون والمستقيم يميلون إلى زيادة خطر الإصابة بسرطان القولون؟ نعم لا لا أعرف 6- هل المدخنون اكثر عرضة للإصابة بسرطان القولون والمستقيم؟ نعم لا لا أعرف 7- هل يميل الأشخاص الذين يشربون الكحول إلى زيادة خطر الإصابة بسرطان القولون والمستقيم؟ نعم لا لا أعرف 8- هل يميل الأشخاص الذين يتناولون الكثير من اللحوم الحمراء إلى زيادة خطر الإصابة بسرطان القولون والمستقيم؟ نعم لا لا أعرف 9- هل يميل كبار السن إلى زيادة خطر الإصابة بسرطان القولون والمستقيم؟ نعم لا لا أعرف ١٠- هل الرجال اكثر عرضة للإصابة بسرطان القولون والمستقيم؟ نعم لا لا أعرف 11- هل الأشخاص الذين يعانون من متلازمة القولون العصبي يميلون إلى زيادة خطر الإصابة بسرطان القولون والمستقيم؟ نعم لا لا أعرف ما هي أعراض سرطان القولون ؟12- وجود دم في البراز. فقدان الوزن المفاجئ زيادة الإفرازات المخاطية في البراز الشعور بعدم إفراغ المستقيم بشكل كامل بالتبرز آلام وتشنجات في المعدة تغير مفاجئ في عدد حركات الأمعاء والإسهال ما هي طرق الكشف المبكر عن سرطان القولون؟ 13- تنظير القولون منظار المستقيم الكشف عن الدم في عينة البراز صبغة الباريوم للأمعاء الغليظة تحاليل الدم الأشعة المقطعية على البطن الفحص السريري للمستقيم ١٤-متى يبدا الانسان بالفحص عند ظهور الاعراض من عمر ٣٥-٤٠ ٤٠-٥٠ ٥٠-٦٠

**Table 9:** Practice items regarding colorectal carcinoma English version modified from [1] and translated into Arabic by the authors.

1-Have you ever searched for information about colorectal cancer prevention intentionally? Yes No If yes what is your source 2-Have you ever taken part in a colorectal cancer screening? Yes No 3-I go to see the doctor if i felt some of the previous symptoms? Yes No Probably 4- How do you intend to get tested if you find symptoms? colonoscopy PR speculum Blood detection in the stool sample Barium dye for large intestine Why you chose this test	١-هل سبق لك أن بحثت عن معلومات حول الوقاية من سرطان القولون والمستقيم عن عمد؟ نعم لا اذا نعم المصدر ٢-هل سبق لك أن شاركت في فحص سرطان القولون والمستقيم؟ نعم لا ٣-أذهب لرؤية الطبيب إذا شعرت ببعض الأعراض السابقة؟ نعم لا من المحتمل ٤- كيف تنوي الفحص ان وجدت أحد الاعراض؟ منظار القولون منظار المستقيم الكشف عن الدم في عينة البراز صبغة الباريوم للأمعاء الغليظة ت ا ولماذا اخترت هذا الفحص

**Table 10:** Attitude items concerning colorectal carcinoma English version modified from [1] and translated into Arabic by the authors.

1-It's important for me to know about colorectal cancer Strongly Agree Agree Neutral Disagree Strongly Disagree 2- Colorectal cancer diagnosed in an early stage can be treated better Strongly Agree Agree Neutral Disagree Strongly Disagree 3-The regular physical examination can find colorectal cancer at an early stage Strongly Agree Agree Neutral Disagree Strongly Disagree 4-Colorectal cancer screening tests are effective Strongly Agree Agree Neutral Disagree Strongly Disagree 5-Cancer screening should be widely implemented Strongly Agree Agree Neutral Disagree Strongly Disagree	1- من المهم بالنسبة لي أن أعرف عن سرطان القولون والمستقيم موافق بشدة يوافق حيادي تعارض لا أوافق بشدة 2- يمكن علاج سرطان القولون والمستقيم الذي يتم تشخيصه في مرحلة مبكرة بشكل أفضل موافق بشدة يوافق حيادي تعارض لا أوافق بشدة 4- اختبارات فحص سرطان القولون والمستقيم فعالة موافق بشدة يوافق حيادي تعارض لا أوافق بشدة 5-يجب تنفيذ فحص السرطان على نطاق واسع موافق بشدة يوافق حيادي تعارض لا أوافق بشدة

**Table 11:** Reasons for not doing colonoscopy English version modified from [6] and translated into Arabic by the authors.

Reasons: *There are no symptoms *Lack of information *Fear or embarrassment of the procedure *Others such as lack of time, difficulty of getting appointment	الأسباب: خيار متعدد *لا توجد أعراض *قلة المعلومات * الخوف أو الإحراج من الإجراء * أخرى مثل ضيق الوقت، وصعوبة الحصول على موعد ا

**Table 12:** Sources of information English version modified from [6] and translated into Arabic by the authors.

Sources of information *Awareness campaign *Booklets *RAD/TV *Newspaper *Doctors *Friends *WHO\ MOH *social media (Twitter, Facebook) *WhatsApp *Internt	مصادر المعلومات *حملة توعية *الكتيبات *راد/تلفزيون *جريدة *الأطباء *أصدقاء *منظمات صحية(الصحة العالمية والسعودية) *وساءل التواصل الاجتماعي(فيسبوك،تويتر) *واتساب *الانترنت
